# Use of Phages to Treat Antimicrobial-Resistant *Salmonella* Infections in Poultry

**DOI:** 10.3390/vetsci9080438

**Published:** 2022-08-18

**Authors:** Md Abu Sayem Khan, Sabita Rezwana Rahman

**Affiliations:** Department of Microbiology, University of Dhaka, Dhaka 1000, Bangladesh

**Keywords:** bacteriophages, *Salmonella*, poultry, biocontrol, drug-resistant

## Abstract

**Simple Summary:**

Salmonellosis, an infection in humans and animals caused by *Salmonella* spp., poses a major concern to public health and food safety worldwide. Antibiotics are mostly prescribed to treat salmonellosis. Unfortunately, indiscriminate use of antibiotics leads to the emergence and transmission of multidrug-resistant *Salmonella* spp. As antibiotics are becoming increasingly ineffective, infections caused by MDR strains will be difficult to manage. The search for an alternative to antibiotics has led scientists to give renewed attention on phage therapy. Though commercial use of phages for controlling *Salmonella* in poultry is still in its early stage, the use of lytic phages is considered an environmentally friendly, cost-effective, and sustainable antimicrobial approach. Moreover, it provides advantages over antibiotics in terms of specificity, cost of development, resistance, and genetic amenability. Studies on laboratory and field scale use show promise on the effectiveness of phages against MDR *Salmonella* spp. However, inadequate data on safety of phage use, phage stability, and lack of regulatory framework remain major obstacles in the commercial application of phages. Our article provides a comprehensive overview on global prevalence and antimicrobial resistance of *Salmonella* in poultry, the efforts to control *Salmonella* using phage therapy, and challenges as well as future prospects of phage therapy.

**Abstract:**

*Salmonellosis* is one of the most common bacterial infections that impacts both human health and poultry production. Although antibiotics are usually recommended for treating *Salmonella* infections, their misuse results in the evolution and spread of multidrug-resistant (MDR) bacteria. To minimize the health and economic burdens associated with antimicrobial resistance, a novel antibacterial strategy that can obliterate pathogens without any adverse effects on humans and animals is urgently required. Therefore, therapeutic supplementation of phages has gained renewed attention because of their unique ability to lyse specific hosts, cost-effective production, environmentally-friendly properties, and other potential advantages over antibiotics. In addition, the safety and efficacy of phage therapy for controlling poultry-associated *Salmonella* have already been proven through experimental studies. Phages can be applied at every stage of poultry production, processing, and distribution through different modes of application. Despite having a few limitations, the optimized and regulated use of phage cocktails may prove to be an effective option to combat infections caused by MDR pathogens in the post-antibiotic era. This article mainly focuses on the occurrence of salmonellosis in poultry and its reduction with the aid of bacteriophages. We particularly discuss the prevalence of *Salmonella* infections in poultry and poultry products; review the trends in antibiotic resistance; and summarize the application, challenges, and prospects of phage therapy in the poultry industry.

## 1. Introduction

*Salmonella*, a rod-shaped, gram-negative, facultative anaerobic, and motile bacterium, is mostly known for its versatile ability to cause a wide spectrum of diseases in humans and animals, such as salmonellosis, typhoid fever, septicemia, and fowl typhoid [1,2]. Based on variations in somatic and flagellar antigens, 2500 serotypes of *Salmonella enterica* have been identified, representing approximately 99% of the pathogenic strains of *Salmonella* [1]. *Salmonella* belongs to the Enterobacteriaceae family and can be acquired from food, water, and environmental sources. It is widely prevalent in the intestinal tract of various animals, such as poultry, cattle, and pets [3]. The transmission of *Salmonella* from animals to humans can occur by both the consumption of food, water contaminated with animal waste, and by direct contact with *Salmonella*-infected animals (fecal–oral route) [4]. *Salmonella* can enter the food chain from animal feed and poultry processing sites, persistently contaminated livestock environments, contaminated hatcheries, vertical transmission, and can be disseminated to humans through the fecal–oral route. In humans, salmonellosis may develop 12–72 h after the consumption of food contaminated with *Salmonella*. It is characterized by fever, diarrhea, vomiting, and abdominal cramps. Globally, three million deaths have been reported among 1.3 billion estimated cases of *Salmonella*-associated gastroenteritis annually [5]. Moreover, the incidence of infections is higher in developing countries, posing a considerable burden to their health and economy [5,6]. In 2018, *Salmonella* was found to be responsible for 33% of the 5146 foodborne outbreaks, resulting in 48,365 illnesses in European Union (EU) member states [7]. The frequent occurrence of *Salmonella* in poultry and poultry products has been identified to be a potential threat to the growth and development of this industry worldwide. In the United States, contaminated poultry and red meats are responsible for one-third of *Salmonella*-associated infections and the annual economic losses have been estimated to be between $2.3 billion and $11.3 billion [8].

Antibiotics are permitted in poultry-producing countries for treating Salmonellosis and promoting growth [9]. Several efforts have been made to minimize antibiotic use in agriculture and poultry production in numerous countries. Sweden imposed a ban on the use of all growth promoting antibiotics in 1986. Denmark also outlawed the use of avoparcin and virginiamycin in 1995 and 1998, respectively. The European Union (EU) banned the use of avoparcin and four other antibiotics (bacitracin, spiramycin, tylosin, and virginiamycin) as growth promoters in 1997 and 1998, respectively [10]. One of the first nations in Africa to outlaw widespread use of antibiotics in livestock was Namibia [11]. In the United States, many antimicrobials were administered to livestock for growth promotion purposes, before the Food and Drug Administration (FDA) made those uses illegal at the start of 2017 [12]. Unfortunately, the misuse of antibiotics leads to the development and transmission of antibiotic-resistant pathogens, rendering antibiotics ineffective in the clinical management of infections. Moreover, resistant determinants can be transferred to other pathogens via horizontal gene transfer mechanisms, thereby promoting resistance in environmental pathogens. As per the ongoing antibiotic resistance trends, poultry production areas serve as an important reservoir of antimicrobial resistance (AMR) genes [13]. Alarmingly, poultry pathogens are now resistant to colistin, which is considered a last-resort drug for treating complicated bacterial infections in humans [14]. Considering the mortality, morbidity, and cost of treatment of diseases caused by multidrug-resistant (MDR) pathogens, different novel approaches are now under investigation to provide a sustainable solution.

Phages are viruses infecting bacteria; they can be classified as lytic (which kill the host at the end of replication) and lysogenic (which integrate their genome into the host genome) [15]. The application of bacteriophages is considered to be an emerging treatment option for preventing bacterial infections in humans and poultry. Because of their enormous bactericidal activity, host specificity, self-limiting capabilities, and ease of genetic manipulation, researchers now consider bacteriophages as potential alternatives to antibiotics [16]. A broad range of bacteria are susceptible to infection by bacteriophages that can tolerate a wide range of temperatures and pH levels. Moreover, high degree of host specificity allows lytic bacteriophage to kill only one species of bacteria. Hence, they are suitable candidates for therapeutic application. Many studies have reported the efficiency of host-specific phages in decreasing bacterial counts in different food items, such as meat, eggs, animal skin, vegetables, and processed foods [17,18,19,20]. The utilization of bacteriophages could be an effective intervention strategy to decrease the colonization of *Salmonella* in animals. Phage administration was found to reduce the *Salmonella* Enteritidis count in experimentally infected broiler chickens by 4.2 log_10_ CFU [21]. Further reduction could be achieved by the appropriate selection of bacteriophages, use of phage cocktails, and optimization of environmental conditions, among others [21]. Given the advantages of bacteriophages over antibiotics, the current explosion in research on the use of these bactericidal viruses in the food and poultry industries has resulted in the development of phage-based products that are now commercially available in developed countries. However, inactivation by harsh environmental conditions (temperature, pH, UV light), cost of large-scale production, and safety issues are major drawbacks that make phage application not suitable for all circumstances in controlling *Salmonella* [22].

## 2. Salmonellosis and Its Association with Poultry 

Although the typical infectious dose of *Salmonella* for causing salmonellosis is 10^7^ to 10^9^ CFU/g, it may vary depending on the composition of foods and the health status of patients [23]. Lower infectious doses are linked to high fat content of the contaminated food products [24]. *Salmonella* Enteritidis has been reported to be the most frequently (65%) encountered serotype in nontyphoidal salmonellosis cases globally, followed by *Salmonella* Typhimurium (12%) and *Salmonella* Newport (4%) [25]. Moreover, *Salmonella* Enteritidis was found to be the dominant serotype in Asia, Latin America, Europe, and Africa, being detected in 38%, 31%, 87%, and 26% of clinical isolates, respectively [25]. *Salmonella* is also responsible for 93.8 million gastroenteritis cases worldwide, resulting in 155,000 deaths annually [26]. In total, 82,694 confirmed cases of salmonellosis were reported in 2013, making it the second most common zoonotic disease in Europe [27]. According to the CDC, *Salmonella* is responsible for approximately 1.35 million illnesses, resulting in 26,500 hospitalizations and 420 deaths annually in the USA [28]. 

Various foods, such as meat, eggs, vegetables, beef, pork, and milk, are often considered to be the major vehicles of *Salmonella* infections in humans [29]. *Salmonella* Enteritidis is typically found in numerous poultry products, whereas *Salmonella* Typhimurium is found in different animals, including pigs, cattle, and poultry [30]. Other serovars that are known to be associated with egg contamination are *Salmonella* Gallinarum, *Salmonella* Pullorum, and *Salmonella* Heidelberg [31]. In the EU, contaminated foodstuffs, especially table eggs and pig meat, act as a source of *Salmonella* infection in humans. However, the risks of consuming broiler and turkey meat are similar and around two-fold lower (EFSA, 2012). Between 1985 and 2002, egg contamination was identified as the primary source, accounting for 53% of *Salmonella* infections reported to the CDC in the United States [32]. *Salmonella* can contaminate eggs by two possible routes. Bacterial infection as a result of fecal material on the eggshell (trans-shell) or exposure during the hatching phase in commercial hatch cabinets, commonly known as horizontal transmission [33,34]. In the second mechanism, known as vertical transmission, *Salmonella* Enteritidis infects the reproductive organs, resulting in the contamination of the yolk, albumen, eggshell membranes, or eggshells before oviposition [35]. Table 1 showed the global prevalence of egg contamination by *Salmonella* spp.

The prevalence of *Salmonella* and predominant serotype in live poultry varies from country to country. *Salmonella* is carried by infected living birds and spreads to other birds by lateral transmission, which occurs mostly through feces, dirt, litter, food, water, dust, and feathers. The overall prevalence of different *Salmonella* serotypes among live birds ranges from 6% to 30% [58,59,60,61]. A study from the Republic of Ireland in 2006 found that 27.3% of 362 broiler flocks were infected with *Salmonella* [60]. In Kagoshima, Japan, 49% of 192 broiler flocks and 7.9% of 3071 cecal samples were found to be positive for *Salmonella* from 2009 to 2012 [62]. In the Shandong province, China, fecal swab analysis determined 12.7% prevalence of *Salmonella* in free range chickens in 2015 [63]. A 32% prevalence of *Salmonella* in cloacal swab was observed in Bangladesh during July 2014 to June 2015 [37]. *Salmonella* Mbandaka, *Salmonella* Infantis, and *Salmonella* Enteritidis were the predominant serotypes in the Republic of Ireland, Japan, and China, respectively. Such results indicate geographical differences in prevalence and serotype diversity of *Salmonella* in chicken flocks.

## 3. Antibiotic-Resistant *Salmonella* in Poultry

Antibiotics are utilized in animal farming at a rate of approximately 8 million kg per year, of which 70% is for nontherapeutic purposes (growth enhancement and disease control). In comparison, antibiotics are used at a rate of only 1.3 million kg per year for treating human infections [64]. Such high use of antibiotics in livestock leads to the emergence of resistant microbes in the native micro-biota of the animal and the local environment due to shedding in the feces [65]. The early development of MDR pathogens resulting from their unrestricted use makes infection management critical. 

A high degree of AMR is frequently observed among *Salmonella* spp. isolated from eggs. Although several antibiotics are used in the poultry production chain, *Salmonella* exhibited the highest resistance to nalidixic acid and ampicillin [66]. Bacterial resistance to tetracycline, oxytetracycline, and nalidixic acid was found to be much higher in commercial layer hen eggs than in duck eggs in India [67]. In Bangladesh, *Salmonella* from chicken egg surfaces exhibited complete resistance to ampicillin and amoxicillin, followed by tetracycline, ciprofloxacin, and colistin [68]. In China, Xie et al. (2019) reported that *Salmonella* from eggs showed varying degrees of resistance to beta-lactam antibiotics, including amoxicillin, cefazolin, penicillin, and piperacillin, followed by aminoglycosides and tetracyclines, such as gentamicin, kanamycin, streptomycin, minocycline, and tetracycline [42]. Mobile genetic elements facilitate the transfer of AMR genes from one pathogen to another and thus cause the transmission of antibiotic resistance. IncA/C plasmids of *Salmonella* have carried genes that confer resistance to different classes of antibiotics such as aminoglycosides, β-lactams, chloramphenicol, trimethoprim, sulfisoxazole, and tetracyclines [69]. In addition, the transmission of antibiotic resistance genes through poultry litter has already been reported [65]. Antimicrobial resistance in *Salmonella* associated with other poultry products are outlined in Table 2.

Infections caused by MDR bacteria result in prolonged hospitalization times, delayed treatment procedures, and increased medical costs. If this situation continues, food security, human health, and biodiversity will be threatened. Changes in husbandry, hygiene, disinfection, monitoring of breeding populations, legal controls, and enforcement of existing regulations have allowed major reductions in antimicrobial drug use for poultry in many parts of the world. However, a new, sustainable, and environmentally-friendly antimicrobial technology is needed to ensure safe health for all by reducing the dependence on antibiotics. 

## 4. Bacteriophages an Alternative to Antibiotics in Controlling Pathogens

Bacteriophages, the most ubiquitous organisms on the earth, are commonly known as viruses of bacteria and archaea [91]. The number of phages in the biosphere (approximately 10^31^) is estimated to be 10-fold higher than the number of bacteria [92,93]. The morphological structure of phages consists of nucleic acids inside a protein coat, and the majority of phages have dsDNA as their nucleic acid [94]. Bacteriophages are classified into two different types based on their replication cycle. Lysogenic or temperate phages usually integrate their nucleic acid into the host DNA and are replicated with succeeding generations of hosts. They do not destroy the host at the end of replication. Lytic or virulent phages attach to the host, introduce their genome into the host genome, replicate with the aid of host replication machinery, assemble, and finally destroy the host cell using a phage-encoded enzyme. Lytic phages are getting renewed attention as a potential solution to the ever-increasing AMR crisis. The main reasons for choosing lytic phages for controlling bacterial pathogens include their enormous bacteriolytic activity and their inability to transduce or transfer genetic elements, which provide advantages over lysogenic phages [91]. Bacteriophages provide some unique advantages that make them an attractive and suitable alternative to antibiotics. First, unlike antibiotics, which have broad-spectrum activity, phages are highly specific to the host; thus, there is less chance of gut dysbiosis and secondary infections following phage therapy. Second, while it takes millions of dollars and a long time to develop a new antibiotic, the isolation, propagation, and large-scale production of phages are less expensive. Third, the other characteristics that make phages more advantageous are their ability to spread through the body upon systemic administration, their ability to cross the blood–brain barrier, and their biofilm inhibitory activity [95,96]. Finally, and most importantly, bacterial resistance to phage therapy is considered less significant in comparison with bacterial resistance to antibiotic therapy. If pathogens develop phage resistance, it is possible to counteract by utilizing modern genetic engineering tools because phages are amenable to genetic manipulation; however, this is quite impossible in the case of antibiotic resistance. Even pan-antimicrobial resistant bacteria are likely to remain fully susceptible to phage attack, provided a suitable phage can be found or with the development of genetically engineered phages.

Current investigations on phage therapy have revealed promising outcomes in treating infections caused by MDR, extensively drug-resistant (XDR), and pandrug-resistant (PDR) bacteria. Infections caused by ESKAPE pathogens (*Enterococcus* spp., *Staphylococcus aureus*, *Klebsiella pneumoniae*, *Acinetobacter baumannii*, *Pseudomonas aeruginosa*, and *Enterobacter* spp.) are currently posing challenges to healthcare management. The application of bacteriophages has been proven to be effective in controlling ESKAPE pathogens [97,98]. The number of XDR *A. baumannii* (XDRAB) populations was found to reduce from 10^8^ to 10^3^ CFU/mL within 30 min of application of the phage φkm18p. The phage also improved the survival rate of lung epithelial cells [99]. Anti-*Salmonella* phage cocktail treatment significantly lowered cecal *Salmonella* concentrations, while simultaneously reducing ileal *Salmonella* contents in swine [100]. Several studies have reported the successful application of bacteriophages in humans to treat septicemia caused by *P. aeruginosa*, prostatitis caused *by E. faecalis*, and MDR *S. aureus*-associated chronic rhinosinusitis [101,102,103]. Phage therapy was found to result in the prevention of infection and improvement of the patients’ condition. Moreover, 6 months following lytic phage treatment (through an eyedrop formulation) in a 65-year-old woman suffering from secondary eye infections caused by vancomycin-resistant *S. aureus* (VRSA), the results of the VRSA culture test were negative [104]. This indicates that phages can be delivered through different routes of administration. Commercially available phage products are now used to overcome bacterial contamination and to cure infections in humans. PhagoBioDerm, a polymeric bandage containing a phage cocktail, ciprofloxacin, and other ingredients, is used to heal wounds caused by *S. aureus* and *P. aeruginosa* [105]. Moreover, treatment with ListShield, a commercially available phage cocktail preparation, was found to reduce *Listeria monocytogenes* contamination in experimentally inoculated frozen entrees, lettuce, smoked salmon, and cheese by 99%, 91%, 90%, and 82%, respectively [106]. It is clear from the abovementioned examples that the optimized use of monophage or phage cocktails in humans, animals, and foods holds great promise to treat infections caused by antibiotic-resistant bacteria and can be a very good alternative to antibiotics. However, phage therapy has several limitations as a potential antibiotic alternative in terms of commercial use. These include lack of experimental procedures for maintaining quality and safety of phage formulation, lack of studies on stability of phage preparation, unclear evidence on the effectiveness on biofilm degradation in serving commercial purpose, development of phage resistance, lack of data on pharmacokinetics and immune response, etc. [107].

## 5. Application of Phages for Controlling *Salmonella* Infections in Poultry and Poultry Products 

In the poultry industry, phage treatment of *Salmonella* serves two fundamental purposes. First, phage treatment minimizes the losses caused by the effects of bacterial pathogens on animal health and production. Second, phage-based biocontrol is considered a powerful tool to control the prevalence of foodborne infections in humans. Selecting the appropriate phages, phage titer, mode of application, and duration of application are the major factors that determine the therapeutic effectiveness of phages [21,108]. Phage cocktails can be administered through different approaches, e.g., by oral administration after mixing with water or as a feed additive, by spraying on eggs, or by direct addition of the phage suspension to contaminated products. Thus, bacteriophages can be a promising intervention strategy to curb the horizontal and vertical transmission of *Salmonella*. The use of phages as an aerosol spray during the transfer of eggs from incubators to hatchers could be a cost-effective way to reduce the horizontal transmission of *Salmonella* via eggs [109]. A study conducted to decrease *Salmonella* colonization in chickens by the oral inoculation of phage preparations have stated that the utilization of phages could pose an effective barrier to the vertical transmission of this pathogen [110]. Among all identified *Salmonella* phages, the most well-known are P22 and Felix-O1. Felix-O1, a broad-spectrum lytic phage, can lyse a wide number of *Salmonella* serotypes and is recognized as an efficient candidate for therapeutic and diagnostic applications [111]. The experimental studies that applied bacteriophage preparations (in monophage or cocktail form) on experimentally infected poultry and poultry products through different mode of administration and the outcomes of phage treatment are outlined in Table 3.

Although small-scale studies have demonstrated a desirable reduction in bacterial counts following phage treatment, its industrial-scale application needs adequate safety assessment. With an increase in the number of studies, more data will be available on the safety and efficacy of phage therapy. In a phage therapy trial including 34,680 broiler chickens at a commercial farm with a previous record of *Salmonella* outbreak, no significant mortality, productivity, and alteration in the gut microbiota were noted in the phage-treated group compared with the untreated control group, indicating the safety of the phage preparation. Here, the effect of phage treatment on gut microbiota was evaluated by 16s rRNA gene amplicon sequencing. So far, this is the largest trial to evaluate the safety and efficacy of *Salmonella* phages in a commercial setting [139,140]. 

## 6. Challenges of Using Phages in Poultry and Probable Solutions

Researchers face several limitations when using phages for the elimination of pathogens. The main challenges associated with the use of phage therapy against *Salmonella* in poultry can be divided into four categories: development of phage resistance in bacteria, selection of the candidate phage, delivery of the phage to the site of infection, and difficulties associated with the regulatory approval of phage products [141]. 

The mechanisms underlying the development of phage resistance in bacteria include host cell surface and extracellular modifications, such as receptor adaptations, outer membrane vesicles, and quorum sensing, as well as intracellular modifications, such as abortive infections, phage exclusion, restriction modification (RM) systems, and CRISPR/Cas systems [142]. RM is the most ubiquitous phage resistance mechanism present in bacteria and archaea; it is also known as the innate immune system of prokaryotes. The RM system identifies host DNA based on the methylation pattern and cleaves foreign DNA [143]. The phage exclusion mechanism, superinfection exclusion system, and abortive infection mechanism prevent phage DNA replication in the host cell and block the entry of phage genetic materials into the host. Thus, phage dissemination becomes limited due to premature bacterial death upon phage infection [144]. However, overcoming phage resistance is not an insurmountable problem because phages have counteracting mechanisms. Phages with the ability to acquire new receptors can change their receptor-binding proteins. Thus, when a host receptor changes to a mutant form, phages can recognize the changing receptor structure and counteract disruptions in phage adsorption receptors. To get around the wide range of RM systems, phages employ various active and passive anti restriction techniques [144].

Potential phage candidates must be virulent and propagate inside the host via the lytic cycle. Phages harboring virulence or AMR genes or carrying integrase or recombinase are not ideal for successful therapeutic applications. According to international experts, an ideal phage cocktail should include phages from different families or groups that have a broad host range, optimum adsorption ability, and the ability to withstand a wide range of physicochemical conditions [145]. Adherence to these criteria must be ensured during the primary phage selection process. Whole-genome sequencing is preferred to provide genomic insights and to confirm that the selected phages are unable to perform transduction and horizontal gene transfer [21,114,141]. Moreover, the incorporation of phages into cocktails boosts their potential for presumed usage. The higher the number of phages present in a certain formulation, the greater the likelihood of its long-term medical and commercial demand [146]. A study assessed four different methods (direct spot test, efficiency of plating, planktonic killing assay, and biofilm assay) to identify the most suitable one for formulating phage cocktails and concluded that the planktonic killing assay is a good choice when considering phage cocktails [147].

Delivering a phage at the site of infection is a major challenge during phage application, especially in live animals. To reduce *Salmonella* colonization on chicken meat, eggshell, or processed food, phage preparations can be sprayed or directly applied to the products. As *Salmonella* initially colonizes the chicken gut, a phage preparation needs to be administered orally to reduce bacterial colonization. In the gut, the phage will encounter acidic pH, resulting in a higher chance of inactivation if it cannot tolerate acidic pH levels. Alternative solutions, such as encapsulation (microencapsulation or liposomal encapsulation), dry formulation, or liquid formulation, can protect phages from acidic conditions [147]. The free phage Felix-O1 was found to be undetectable after 5 min of exposure to pH 3.7 because this phage is highly sensitive to acidic pH. However, this problem was overcome by delivering microencapsulated Felix-O1 through a chitosan–alginate–CaCl2 system that kept the phage viable for 1 h in simulated gastric juice (pH 2.4) and for 3 h in porcine bile extract [148].

For the commercialization of phages, specific regulatory pathways are necessary depending on their use as feed additives, disinfectants, or medicines. Developers should go through regulatory routes and present adequate data on the safety and efficacy of the products before marketing. It is obvious to establish basic safety issues to ensure confidence in using phages as antimicrobials. Examples of such issues include the impact of phage on microbiome, bacterial lysis-associated endotoxin release, immune and inflammatory response, biological and chemical contaminants in phage preparations, and others [149]. Adoption of a specific framework addressing safety criteria, safety endpoints, methods of safety assessments, quality assurance of phage preparations, etc., will advance commercialization of phage application. Besides, the regulatory issue related to phage therapy affects not only the market placement of phage-based products but also the conduct of clinical trials [150]. A significant obstacle to the veterinary use of phages in the EU is that bacteriophages do not fit into the existing EU regulations regarding the use of feed additives [151]. The EU found the current regulatory framework quite unsatisfactory and is looking for national solutions for the satisfactory regulation of phage therapy. The Food and Drug Administration (FDA) regulates phages in the United States, regardless of whether they are to be used in humans or animals. However, they go through distinct stages depending on how they will be utilized [141]. Nevertheless, the regulatory framework will undergo substantial changes in the future to ease the way of using phages for combatting MDR pathogens in poultry and poultry products, and to maintain adequate safety measures.

## 7. Future Prospect of Bacteriophage 

Undoubtedly, phage therapy has huge potential in future medicine to tackle antibiotic resistance in humans, animals, and agriculture. The growing interest in phages as food antimicrobials has prompted more research on the efficiency of single or mixed phages against target bacteria while posing minimum concerns to human health. The bactericidal activity of phages and their advantages over antibiotics rapidly expand the research and development of introducing bacteriophage-based novel products into the global market. Approval and commercialization of AgriPhage developed by Omnilytics Inc(Sandy, UT, United States) for agricultural uses, EcoShield, and SalmoFresh by Intralytix Inc(Baltimore, MD, United States). to use against *E. coli* O157:H7 and *Salmonella* spp. occurring in ready-to-eat foods, poultry, and poultry products are examples of such development [152]. Synergistic application of phage and antibiotics, co-administration of phages with enzymes, genetic modification of phage to improve phage therapy outcomes, and utilizing engineered phages to deliver drugs are the emerging areas of phage therapy research that will bring substantial changes in medical and veterinary therapeutics. 

**Figure 1:**
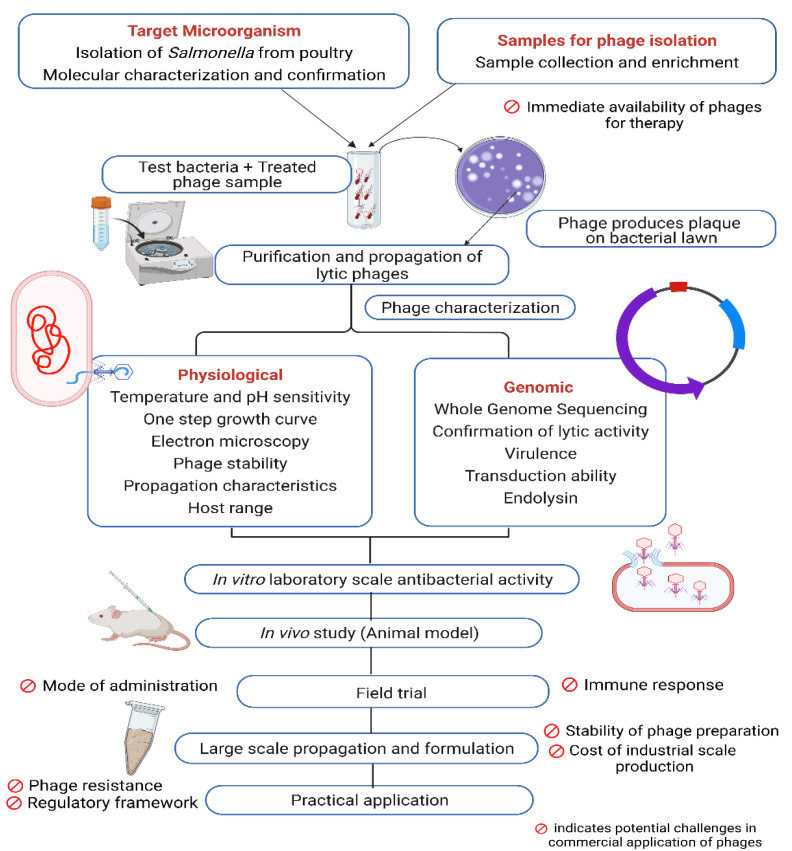
Steps in phage application process with potential challenges in poultry industry.

Antimicrobial resistance is more prevalent in developing nations due to inadequate infrastructure for healthcare, maintaining unregulated process in agricultural production, poor sanitation and hygiene, and widespread antibiotic overuse. Alternative treatment modalities are critically needed in the developing world due to public health and antibiotic resistance issues and, for various reasons, phage therapy has the potential to address the crisis. Lytic phages destroy specific host bacteria without harming gut microbes and eukaryotic cells. The ubiquity of phages keeps them available in wastewater, sewage, and excreta for isolation. Finally, the fast development, cost effectiveness, and environmentally-friendly characteristics of phage products make this a well-suited strategy to fight against MDR bacteria in developing countries [153]. Developing countries can also benefit economically from phage-mediated control of infectious diseases. Bacterial infections in poultry, cattle, and livestock result in huge financial losses each year. Moreover, the physiological and genetic makeup of causative agents vary between regions. Developing phage cocktails against local strains of bacteria may contribute to reduction in infections, thus decreasing economic losses. Establishment of phage-oriented biotech industries in those countries can address global crises by producing novel bio products, creating more job opportunities, and also helping to compete in a global market with new solutions to veterinary infectious diseases. A general procedure from isolation to application of phages to control *Salmonella* in poultry is illustrated in Figure 1.

## 8. Conclusions

The identification of a new class of antimicrobials is of utmost importance to protect public health from the devastating effects of AMR. The emergence and transmission of antibiotic-resistant pathogens have opened a new window for phage therapy, which has a long history of use since its discovery. Current studies on phage therapy to reduce the prevalence of *Salmonella* in poultry have revealed promising outcomes that promote the development and use of bacteriophage-based products, not only to prevent the misuse of antibiotics but also to ensure food safety for the global population. Recent advancements in the fields of genomics and proteomics can help overcome the obstacles related to safety issues associated with the use of phages in food and animal production. Unlike antibiotics, with the current progression of phage research, the propagation, manipulation, and commercial-scale use of host-specific bacteriophages could serve as a sustainable technology that would drastically change the scenario and impact of AMR in the poultry industry, especially in developing countries.

## Figures and Tables

**Table 1:** Prevalence of *Salmonella* spp. in eggs in different countries.

Country	Prevalence	Sample	Year	References
India	4.82%	Eggs	2006–2007	[36]
Bangladesh	28% and 83%, 3%	Eggs and eggshell, egg content	2014–2015 and 2011–2012	[37,38]
Ethiopia	2.4%, 4.8%, and 5.3%	Egg content, eggshell and egg from market	2018 and 2012–2013	[39,40]
China	6.6%, 5.5%	Eggs from poultry, eggs from marketplace	2013–2014	[41]
Guangdong, China	5.4%	Eggs	2017–2018	[42]
Iran	13.61%	Eggshell	1996–2018	[43]
Pakistan	29.36% and 10.31%	Eggshell and egg content	2011–2012	[44]
Kuwait	10%	Eggs	2004–2005	[45]
Iraq	4.85%	Eggshell	2016	[46]
Thailand	12.4%, 11%	Eggshell, egg content	1992	[47]
Nigeria	7.3%	Eggs	2019	[48]
Brazil	1.25%	Eggshell, egg content	n/a	[49]
Sri Lanka	6.7%	Eggs	n/a	[50]
Zambia	2.31%	Eggshell	2018	[51]
Uruguay	0.0049%	Egg content	2010	[52]
Japan	0.25%	Eggshell	2007–2008	[53]
South Korea	7.4%	Egg content	2010–2012	[54]
Colombia	2.93%	Eggshell	2014	[55]
Cameroon	88.6%	Eggs	2016	[56]
Ireland	0.04%	Egg contents	2005–2006	[57]

**Table 2:** Summary of the results of studies on antibiotic resistance among *Salmonella* isolates in poultry and poultry products in the last decades.

Origin	Sample Type	Dominant Serotype	Phenotypic Resistance	Reference
Bangladesh	Cloacal swab, feed, litter	*Salmonella* Typhimurium	The percentage of resistance to tetracycline, chloramphenicol, ampicillin, and streptomycin were 97.14%, 94.28%, 82.85%, and 77.14%, respectively.	[70]
Bangladesh	Chicken samples (liver and intestine)	*Salmonella* spp.	High percentage of resistance were found against colistin (92.68%) and ciprofloxacin (73.17%), followed by tigecycline (62.20%), co-trimoxazole (60.98%).	[71]
Henan, China	Dead chicken	*Salmonella* Pullorum, *Salmonella* Enteritidis	77%, 73%, 5.60% of isolates were resistant to ciprofloxacin, sulfisoxazole, and ampicillin, respectively; 69.64% were resistant to three or more antimicrobials.	[72]
Egypt	Broiler chicken	*Salmonella* Enteritidis, *Salmonella* Typhimurium	76.7% isolates were multidrug resistant, resistant to sulfamethoxazole (100%), amoxicillin–clavulanic acid (68%), streptomycin (65%).	[73]
Iran	Fecal swab	*Salmonella* Enteritidis	Resistant to nitrofurantoin (90.2%), followed by nalidixic acid (67.2%), and cephalexin (37.7%). Multi-drug resistance characteristics were found in 57.4% isolates.	[74]
Pakistan	Poultry postmortem	*Salmonella* Infantis	Isolates showed maximum resistance against pefloxacin (94.4%), chloramphenicol (83.3%), and imipenem (77.7%).	[75]
Eastern region, China	Fecal swab	*Salmonella* Indiana, *Salmonella* Enteritidis	Isolates were resistant to sulfamethoxazole, ampicillin, tetracycline, doxycycline, and trimethoprim.	[76]
South Korea	Chicken meat, feces, and eggshells	*Salmonella* Enteritidis	All isolates were found to be resistant to at least 1 of 21 antibiotics, 65.2% were resistant to three or more antimicrobials, namely penicillins, sulfisoxazole, streptomycin, tetracyclines, quinolones.	[77]
India	Eggs, cloacal swabs, feces	Salmonella Typhimurium	All the isolates showed resistance to clindamycin, oxacillin, penicillin, and vancomycin at varying degree.	[78]
Romania	Chicken meat	*Salmonella* Infantis	66.6% of isolates were resistant to tetracycline, followed by nalidixic acid and sulfamethoxazole (64.3%), ciprofloxacin (61.9%), streptomycin (59.5%).	[79]
South Africa	Chicken carcass swabs, cloacal swabs	*Salmonella* Bovismorbificans, *Salmonella* Hadar, *Salmonella* Dublin, *Salmonella* Enteritidis	The frequency of MDR among the *Salmonella* isolates was 81.8%, highest to erythromycin (94.9%) and spectinomycin (82.7%).	[80]
Malaysia	Cloaca swab	*Salmonella* spp.	Resistance to erythromycin (100%), chloramphenicol (76.2%), tetracycline (62%), ampicillin (47.7%), sulfamethoxazole/trimethoprim (42.9%).	[81]
Thailand	Chicken meat	*Salmonella* spp.	Nalidixic acid had the highest rate of resistance (31%), followed by ampicillin (24%), tetracycline (19%), and sulfamethoxazole trimethoprim (8%).	[82]
Greece	Chicken carcass and liver	*Salmonella* Hadar, *Salmonella* Enteritidis,	The percentage of resistance to streptomycin, tetracycline, nalidixic acid, ampicillin, and rifampicin were 64.5%, 56.2%, 39.5%, and 33.3%, respectively.	[83]
Vietnam	Chicken carcasses	*Salmonella* Albany,	73.3% isolates were resistant to at least one antibiotic with highest resistance to tetracycline (59.1%) and ampicillin (41.6%); 17.7% multidrug-resistance was also observed.	[84]
Singapore	Chicken meat	*Salmonella* Saintpaul	59.6% isolates were multidrug-resistant. Phenotypic resistance to ampicillin, tetracycline and chloramphenicol, sulfamethoxazole-trimethoprim and nalidixic acid were 78.8%, 61.5%, 55.8%, and 30.5%, respectively.	[85]
Colombia	Chicken carcasses	*Salmonella* Paratyphi B	The percentage of *Salmonella* isolates resistant to 1–5, 6–10, and 11–15 antimicrobial agents were 35.2%, 24.6%, and 33.9%, respectively.	[86]
Turkey	Chicken meat	*Salmonella* spp.	High degree of resistance (≥89.2) to vancomycin, tetracycline, streptomycin was observed.	[87]
Myanmar	Chicken meat	*Salmonella* Albany	52.2% isolates were multidrug-resistant. High frequency of resistance to trimethoprim-sulfamethoxazole (70.3%), tetracycline (54.3%), streptomycin (49.3%), ampicillin (47.1%) was found.	[88]
Canada	Chicken meat	*Salmonella* Hadar	About 21% of chicken isolates were resistant to amoxicillin–clavulanic acid, ceftiofur, and ceftriaxone.	[89]
Argentina	Chicken liver	*Salmonella* Schwarzengrund	All isolates were found to be sensitive to all tested antibiotics except 100% resistant to erythromycin.	[90]

**Table 3:** Summary of the experimental studies on phage treatment to reduce colonization of *Salmonella* spp. in poultry and poultry products.

Experimental Model	Phage	Inoculation Dose	Phage Delivery Method	Outcomes (Compared with Control)	Reference
Broiler chicken	Three *Salmonella* phage	10^9–11^ PFU	Oral	Phage reduced cecal colonization of *Salmonella* Enteritidis and *Salmonella* Typhimurium by ≥4.2 log10 CFU and ≥2.9 log10 CFU, respectively, within 24 h.	[21]
Leghorn chicken specific-pathogen-free (SPF)	Three-phage cocktail	10^10^ PFU	Oral	When the bacteriophage cocktail was given 1 day before or immediately after bacterial infection, and then again on different days following infection, there was a decrease in *Salmonella* concentration in the chicken cecum.	[112]
Broiler chicken	Three-phage cocktail	10^11^ PFU	Oral	The colony-forming units of *Salmonella* Enteritidis PT4 per gram of cecal content were reduced by 3.5 orders of magnitude in the bacteriophage-treated group.	[113]
Chicken carcasses	*Salmonella* spp. phage	10⁹ PFU/mL	Spraying	No *Salmonella* Enteritidis was detected in two trials and more than 70% reduction was achieved in the other two trials.	[114]
SPF chicks	*Salmonella* spp. phage	1.18 × 10^11^ PFU–1.03 × 10^2^ PFU	Oral	Cecal contents indicated a moderate decrease in *Salmonella* loads at 3 days post infection (dpi), with a greater reduction at 5 days post infection (dpi). All of the chicks were negative for *Salmonella* from 7 dpi through the completion of the trial at 15 dpi.	[115]
Broiler chicks	Mixture of bacteriophage	2.5 × 10^9^–7.5 × 10^9^ PFU	Oral	Compared to untreated controls, *Salmonella* Enteritidis retrieved from cecal contents was reduced at 12 and 24 h following treatment.	[116]
One-day-old chicks	Bacteriophage ΦCJ07	10^5^, 10^7^ and 10^9^ PFU	Oral	In challenged and contact chickens, all treatments reduced intestinal *Salmonella* colonization; after 3 weeks of treatment, no intestinal *Salmonella* was detected in 70% of contact hens treated with 10^9^ PFU/g of bacteriophage.	[117]
Seven-day old chickens	Three different *Salmonella*-specific bacteriophages	10^3^ PFU	Spray	When competitive exclusion plus bacteriophage was used, the mean *Salmonella* Enteritidis cecal count decreased (1.6 × 10^2^ CFU/g) compared to the control group (1.56 × 10^5^ CFU/g).	[118]
Six-week-old chickens	*Salmonella* Gallinarum (SG)-specific bacteriophage	10^6^ PFU	Oral	In comparison to untreated contact hens, contact hens treated with the bacteriophage showed a considerable reduction in mortality.	[119]
Broiler chicks	*Salmonella* Enteritidis phage	10^8^ PFU	Oral	On day of trial 14, bacteriophage treatments significantly reduced the incidence of *Salmonella* Enteritidis in cloacal swabs.	[120]
Broiler chicks	P22hc-2, cPII and cI-7 and Felix 0	5 × 10^11^ PFU	Oral	In phage-treated hens, average cecal bacterial counts were 0.3–1.3 orders of magnitude lower than in untreated controls.	[121]
Ten-day old chickens	Three lytic phages	10^3^ PFU	Spray and Oral	Aerosol-spray of bacteriophages resulted in 72.7% decrease in the incidence of *Salmonella* Enteritidis infection. In addition, counts of *Salmonella* Enteritidis indicated that phage administration by coarse spray and drinking water decreased the bacteria′s colonization in the gut.	[122]
White Leghorn chicks	Φ st1	10^12^ PFU/mL	Intracloacal inoculation	Within 6 h of post-challenge, the *Salmonella* count had dropped to 2.9 log_10_ CFU/mL, and *Salmonella* Typhimurium was undetectable at and after 24 h.	[123]
Eggs	PSE5	4 × 10^7^ PFU	Immersion	A reduction by 2 × 10^6^ CFU/mL of *Salmonella* was achieved after phage treatment.	[110]
Liquid egg	Pu20	10^8^ or 10^9^ PFU/mL	Direct inoculation	At 4 °C and 25 °C for 24 h, the quantity of live bacteria in the treatment group reduced by up to 1.06 log_10_ CFU/mL and 1.12 log_10_ CFU/mL, respectively, and the highest antibacterial efficacy was 91.30% and 92.40%, respectively, when multiplicity of infection (MOI) = 1000.	[124]
Liquid whole egg	Two phages (OSY-STA and OSY-SHC)	n/a	Direct inoculation	1.8 and >2.5 log CFU/mL reduction in *Salmonella* Typhimurium and *Salmonella* Enteritidis, respectively.	[125]
Chicken breasts and fresh eggs	UAB_Phi 20, UAB_Phi78, and UAB_Phi87	10^9^ PFU/mL and 1 × 10^10^ PFU	Soaking in suspension and spraying	*Salmonella* reduction was >1 log_10_ CFU/g in chicken breasts. In fresh eggs, a reduction of 0.9 log_10_ CFU/cm^2^ in *Salmonella* was observed.	[126]
Raw chicken breast	Five *Salmonella* phages	3 × 10^8^ PFU	Suspension added on surface	The largest reductions in the number of *Salmonella* Enteritidis and *Salmonella* Typhimurium in phage-treated group were 3.06 and 2.21 log CFU/piece, respectively, when incubated at 25 °C.	[127]
Chicken breast	Two-phage cocktails	4 × 10^9^ PFU/mL	Added on surface	After 5 h, the *Salmonella* Enteritidis concentration on chicken breast was reduced by 2.5 log CFU/sample	[128]
Chicken breasts	SPHG1 and SPHG3	8.3 log_10_ PFU	Spotted	The phage cocktail was applied to chicken breasts at MOIs of 1000 or 100, and the viable count of *Salmonella* Typhimurium was significantly reduced.	[129]
Chicken breast meat	Four *Salmonella* phage	10^8^, 10^9^, and 10^10^ PFU/mL	Directly added	When raw chicken breast samples were treated with a cocktail of all four bacteriophages at 4 °C for 7 days, viable cell counts of bacteria were considerably reduced.	[130]
Chicken breast fillets	*Salmonella* lytic bacteriophage preparation	10^9^ PFU/ml	Spraying	*Salmonella* reductions of 1.6–1.7 and 2.2–2.5 log CFU/cm^2^ were achieved with the use of chlorine and PAA followed by phage spray.	[131]
Chicken skin	Eϕ151, Tϕ7 phage suspension	10^9^ PFU	Spray	*Salmonella* reductions were 1.38 log_10_ MPN (Enteritidis) and 1.83 log_10_ MPN (Typhimurium) per skin area following phage treatment.	[17]
Chicken skin	vB_StyS-LmqsSP1	2.5 × 10^8^ PFU/cm^2^	Direct addition	Phage treatment of chicken skin resulted in about 2 log units reduction in *Salmonella* isolates from the first 3 h throughout a 1-week experiment at 4 ℃.	[132]
Raw chicken meat and chicken skin	SE-P3, P16, P37, and P47	10⁹ PFU	Direct inoculation	Throughout storage at 4 and 25 °C, phages reduced the number of viable *Salmonella* cells in samples containing 10^3^ CFU/g to undetectable levels.	[133]
Chicken meat	Five bacteriophages	10^9^ PFU/mL	Direct inoculation	Compared to control, application of phage cocktail results in 1.4 logarithmic unit reduction at 10 ℃ at 48 h.	[134]
Chicken meat	Three lytic bacteriophages Ic_pst11, Is_pst22, and Is_pst24	10^8^, 10^7^, and 10^6^ PFU/mL	Direct addition	At MOIs of 100, 1000, and 10,000, a substantial decline in the viable count of *Salmonella* Typhimurium was seen at 7 h after phage application with reductions of 1.17, 1.26, and 1.31 log_10_ CFU/g.	[135]
Chicken meat	STGO-35-1	4 × 10^6^ PFU/mL	Direct addition	Phage treatment caused a significant 2.5 log_10_ reduction of *Salmonella* Enteritidis.	[136]
Chicken frankfurters	Felix O1	5.25 × 10^6^ PFU	Direct addition of liquid	Suppression levels of 1.8 and 2.1 log units of *Salmonella* Typhimurium were achieved by two variants of phages.	[137]
Duck meat	fmb-p1	9.9 × 10^9^ PFU	Direct inoculation	4.52 log CFU/cm^2^ reduction in *Salmonella* Typhimurium counts in ready-to-eat duck meat was found.	[138]

## Data Availability

Data sharing not applicable.
